# Fluorescently Labeled Gadolinium Ferrate/Trigadolinium Pentairon(III) Oxide Nanoparticles: Synthesis, Characterization, In Vivo Biodistribution, and Application for Visualization of Myocardial Ischemia–Reperfusion Injury

**DOI:** 10.3390/ma15113832

**Published:** 2022-05-27

**Authors:** Dmitry V. Korolev, Galina A. Shulmeyster, Maria S. Istomina, Natalia V. Evreinova, Ilia V. Aleksandrov, Aleksandr S. Krasichkov, Viktor N. Postnov, Michael M. Galagudza

**Affiliations:** 1 Institute of Experimental Medicine, Almazov National Medical Research Centre, Saint-Petersburg 197341, Russia; korolev_dv@almazovcentre.ru (D.V.K.); g.schulmeister@yandex.ru (G.A.S.); ist_mary@mail.ru (M.S.I.); zna47@lti-gti.ru (N.V.E.); postnovvn@mail.ru (V.N.P.); galagudza_mm@almazovcentre.ru (M.M.G.); 2 Laboratory of Blood Circulation Biophysics, Pavlov First Saint-Petersburg State Medical University, Saint-Petersburg 197022, Russia; 3 Department of Nano- and Microelectronics, Saint-Petersburg Electrotechnical University “LETI”, Saint-Petersburg 197376, Russia; 4 Department of Electrochemical Production, Saint-Petersburg State Institute of Technology, Saint-Petersburg 190013, Russia; 5 Department of Radio Engineering Systems, Saint-Petersburg Electrotechnical University “LETI”, Saint-Petersburg 197376, Russia; krass33@mail.ru; 6 Institute of Chemistry, Saint-Petersburg State University, Saint-Petersburg 199034, Russia; 7 Department of Pathophysiology with Clinical Pathophysiology Course, Pavlov First Saint-Petersburg State Medical University, Saint-Petersburg 197022, Russia

**Keywords:** nanoparticles, gadolinium ferrate, trigadolinium pentairon(III) oxide, fluorophores, indocyanine green, biodistribution, myocardial ischemia–reperfusion injury

## Abstract

Various gadolinium compounds have been proposed as contrasting agents for magnetic resonance imaging (MRI). In this study, we suggested a new synthesis method of gadolinium ferrate/trigadolinium pentairon(III) oxide nanoparticles (GF/TPO NPs). The specific surface area of gadolinium ferrate (GdFeO_3_) and trigadolinium pentairon(III) oxide (Gd_3_Fe_5_O_12_) nanoparticles was equal to 42 and 66 m^2^/g, respectively. The X-ray diffraction analysis confirmed that the synthesized substances were GdFeO_3_ and Gd_3_Fe_5_O_12_. The gadolinium content in the samples was close to the theoretically calculated value. The free gadolinium content was negligible. Biodistribution of the GF/TPO NPs was studied in rats by fluorescent imaging and Fe^2+^/Fe^3+^ quantification demonstrating predominant accumulation in such organs as lung, kidney, and liver. We showed in the in vivo rat model of myocardial ischemia–reperfusion injury that GF/TPO NPs are able to target the area of myocardial infarction as evidenced by the significantly greater level of fluorescence. In perspective, the use of fluorescently labeled GF/TPO NPs in multimodal imaging may provide basis for high-resolution 3D reconstruction of the infarcted heart, thereby serving as unique theranostic platform.

## 1. Introduction

To date, various gadolinium compounds have been proposed as contrasting agents for magnetic resonance imaging (MRI). In particular, a new method of synthesis of gadolinium chelates was proposed for the visualization of inflammation [1]. In this study, synthesized gadolinium conjugate was selectively phagocytosed by active macrophages in the synovial membrane of rats with a rheumatoid arthritis model, providing rationale for its use as a targeted MRI contrast agent for the visualization of peripheral inflammation. In order to enhance the T1 MRI contrasting effect, a synthesis method based on wet chemistry was proposed to produce polyacrylic acid-stabilized gadolinium oxide nanoparticles with an average size of 2 nm [2]. In the other study, two generations of dendrons (G–0 and G–1) with NH_2_ peripheral groups have been developed for the conjugation of 1,4,7,10–tetraazacyclododecane–1,4,7, a derivative of triacetic acid and chelation with paramagnetic Gd^3+^ ions [3]. These complexes have a longer T1 MRI relaxation time due to a slower rotation of the molecule as compared to monomeric Gd(III) complexes previously used as contrast agents; therefore, the number of paramagnetic centers present in one molecular structure is increased.

Aouidat F. et al. [4] first reported a new synthesis scheme of Gd(III)–biopolymer–Au(III) complex, which acts as a key ingredient of core–shell gold nanoparticles (Gd @ AuNPs). It was demonstrated that Gd @ AuNPs offer an advantage in hepatocyte visualization in the liver. In particular, these nanoconjugates were characterized by intensive cellular uptake of Gd @ AuNPs while preserving the T1 contrast inside cells, which provides a robust in vivo detection system using T1 MRI. The study of Jiang W. et al. [5] described new bifunctional (MRI)/fluorescent probes obtained by loading gadodiamide into fluorescent silica (NP Gd @ Cy5.5 @ SiO_2_–PEG–Ab) nanoparticles for targeting prostate cancer (PCa). It was shown that the synthesized NP Gd @ Cy5.5 @ SiO_2_–PEG–Ab have a high potential as contrasting agents for MRI/fluorescent identification of prostate-specific membrane antigen-positive PCa cells. In the study of Jiang W. et al. [6], gadopentetic acid-doped silica (GA) (Gd @ SiO_2_) was first synthesized by the reverse microemulsion method, which was followed sequential engraftment of amino and carboxyl groups onto the Gd @ SiO_2_ surface. After that, monoclonal antibodies to the prostate-specific membrane antigen were conjugated with carboxyl-modified Gd @ SiO_2_–COOH nanoparticles by the carbodiimide method. Using this approach, PCa cell-specific nanoparticles for MR contrasting were obtained for PCa imaging. Of note, reverse microemulsion polymerization method was also used to coat iron oxide (IO) nanoparticles with a reconstructed silica shell and to form cores from NP IO @ SiO_2_ [7]. After that, gadolinium chelate was applied to the surface of the cores coated with silica gel. Based on IO @ SiO_2_–DTPA–Gd, a contrast agent was developed and tested both using tumor cell culture and in vivo murine model, demonstrating excellent ability to detect tumor cells using T1 MRI. In the other study, bombesin (BBN)-modified gadolinium oxide nanoprobe (Gd_2_O_3_) containing fluorescein (Gd_2_O_3_–5(6)–carboxyfluorescein [FI]–polyethylene glycol [PEG]–BBN) was synthesized for MRI/fluorescent dual-modality PCa imaging [8]. In this case, bombesin known as a gastrin-releasing peptide analogue specifically binding to GRP receptors that are overexpressed in PCa cells, has been used as a targeting ligand. A liver-targeting MRI nanoparticle contrast agent was developed by conjugation of gadolinium (Gd) chelate groups onto the biocompatible poly(l–lactide)–block–poly (ethylene glycol) (PLA–PEG) nanoparticles [9]. PLA–PEG conjugated with diethylenetriaminepentaacetic acid (DTPA) was used to prepare PLA–PEG–DTPA nanoparticles by solvent diffusion method, and then Gd was loaded onto nanoparticles by chelation with unfolding DTPA on the surface of PLA–PEG–DTPA nanoparticles. In some studies, Gd-containing contrasting agents were produced on the basis of mesoporous silica nanoparticles (MSNs). For instance, MSNs were co-doped with Gd^3+^ and Al^3+^ and then loaded with tris(bipyridine)ruthenium(II) (Ru) by ion exchange to obtain Ru/Gd–Al @ MSN [10]. The prepared nanoparticles were used as contrast agents for in vivo dual modality fluorescent/MRI imaging in mice. Furthermore, mesoporous SiO_2_, Gd_2_O_3_ @ SiO_2_ nanoparticles were synthesized with a new one-step synthesis method to be used as an effective contrast agent for MRI [11]. The prepared nanoparticles were coated with poly lactic–co–glycolic acid (PLGA). Significant enhancement of T1 MRI contrast was found in xenograft tumors of the nasopharyngeal carcinoma CNE–2. Novel multifunctional polymeric nanoparticle contrast agent made of (anti–VEGF PLA–PEG–PLL–Gd NP), simultaneously modified with gadolinium–diethylenetriamine–pentaacetic acid (Gd–DTPA) and anti–vascular endothelial growth factor (VEGF) antibody has been developed for delivery of Gd–DTPA to the tumor area and for achieving the early diagnosis of hepatocellular carcinoma [12]. The anti–VEGF PLA–PEG–PLL–Gd NP exhibited high T1 relaxation and no obvious cytotoxicity under experimental concentrations in human hepatocellular carcinoma (HepG2) cells.

A well-known method of obtaining Gd nanoparticles is evaporation in a flow of pure helium. Such nanoparticles with a diameter of 18–89 nm were obtained in a helium flow with the addition of 0.5% oxygen [13]. It was found that the addition of O_2_ did not affect the particle size, structure, and Curie temperature, although it reduced the magnetization. The 18 nm particles were arranged in a lattice with cubic symmetry and remained paramagnetic below Curie point (T_c_). With an increase in the nanoparticle size, the hexagonal phase proportion increased, which coincided with the gadolinium structure, and below T_c_ such particles became ferromagnetic.

A method of mechanosynthesis of trigadolinium pentairon(III) oxide [14] was proposed by high-energy ball milling of stoichiometry amounts of α–Fe_2_O_3_ and Gd_2_O_3_, followed by short thermal annealing conducted at moderate temperatures. The results of X-ray diffraction and Mossbauer spectroscopy showed the formation of the GdFeO_3_ perovskite phase in the milled samples in relative amounts depending on the milling time. The formation of Gd_3_Fe_5_O_12_ was observed in samples with an annealing temperature of 1000–1100 °C. Other methods of GdFeO_3_ nanoparticle synthesis have also been described in the literature. For instance, GdFeO_3_ nanoparticles were reported to be synthesized by the method of reverse co-precipitation of gadolinium and iron(III) hydroxides from nitrate salts followed by subsequent heat treatment in air [15,16]. After heating at a temperature of 750 °C for 4 h in air, the obtained gadolinium ferrate nanoparticles were transferred in a colloidal solution by ultrasonic treatment of their aqueous suspension.

For several decades, gadolinium chelate-based T1 contrasting agents for MRI were most commonly used clinically [17]. However, owing to prolonged persistence in the organism and nephrotoxicity these agents were not safe enough, leading to the FDA claiming against their clinical use. This situation has stimulated the development of novel gadolinium compounds in the form of stable complexes capable of bioelimination in the intact state [17,18]. The synthesis of such complexes is usually low-yield and, in addition, requires multi-step synthetic approaches [18]. Despite better contrasting effect and lower toxicity of these second-generation gadolinium compounds, compared to gadolinium chelates [19], their synthesis still necessitates the use of unsafe solvents requiring complex and expensive purification [20]. The information about the techniques of gadolinium ferrites synthesis is scarce. Available literature describes the use of gadolinium-doped ferrites of other elements, for example, cobalt [21] and manganese zinc [22], which might again bring some deal of toxicity during in vivo application. It is also known that the existing techniques of gadolinium ferrite manufacturing may not ensure its appropriate purity, thereby necessitating the use of special envelops [23]. The evidence summarized above has stimulated us to develop and describe a new method of gadolinium ferrite synthesis that results in the amount of free gadolinium being nearly zero. In addition, gadolinium ferrite nanoparticles were covered by a simple envelope in order to facilitate fluorescent labelling and the in vivo tracking of nanoparticles.

In this work, we propose a new method for the synthesis of gadolinium ferrate and trigadolinium pentairon(III) oxide nanoparticles (GF/TPO NPs), as well as their modification with (3–aminopropyl) triethoxysilane (APTES), followed by immobilization of the fluorescent dye indocyanine green (ICG). The physicochemical characterization of GF/TPO NPs was performed, followed by the investigation of their biodistribution and assessment of their targeting properties towards ischemic-reperfused myocardium.

## 2. Materials and Methods

### 2.1. Chemicals and Synthesis Scheme

All chemicals were purchased from Sigma-Aldrich (St. Louis, MO, USA). The GF/TPO NP synthesis was carried out in a 250 mL heat-resistant glass beaker. The beaker was placed on a magnetic stirrer. 100 mL of distilled water, gadolinium(III) nitrate, iron(II) sulfate, iron(III) sulfate and ammonium citrate were added to the reaction mixture in the ratio shown in Table 1. The ratios were calculated for a stoichiometric mixture, from which gadolinium ferrate (o–GdFeO_3_) or trigadolinium pentairon(III) oxide (c–Gd_3_Fe_5_O_12_) were expected to be obtained.

The reagents were introduced into the reaction mixture in the sequence indicated in the Table 1. Dissolution of a subsequent reagent was carried out only after complete dissolution of the previous one.

**Figure 1:**
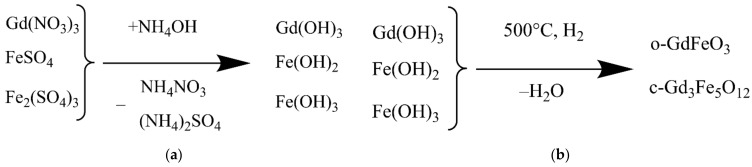
Synthesis scheme: (**a**) deposition of a mixture of hydroxides; (**b**) Synthesis of gadolinium ferrate and trigadolinium pentairon(III) oxide during heating.

After dissolution of all the reagents under constant stirring ammonium hydroxide in a concentration of 12.5 wt% with a volumetric flow rate of 2 mL/min was poured into the reaction mixture using a micro peristaltic pump until the pH value of 7.0–8.0 was achieved. The reaction resulted in the deposition of a mixture of iron(II), iron(III) and gadolinium(III) hydroxides (Figure 1a).

Then the resulting mixture was lyophilized on a VaCo 2 freeze dryer (ZirBus, Bad Grund, Germany) at a temperature of −50 °C under the pressure of 3 Pa for 48 h and mineralized in hydrogen atmosphere at a temperature of 500 °C for 30 min. The synthesis scheme is shown in Figure 1b.

### 2.2. Assessment of GF/TPO NP Physicochemical Properties

Quantification of crystalline phases in the samples was performed using an automated powder diffractometer D2 Phaser (Bruker), X-ray tube radiation was investigated on CuKα1+2, tube operating mode was 30 kV/10 mA, position-sensitive detector, reflection geometry, Bregg–Brentano focusing scheme, T = 25 °C, air atmosphere.

The phases were identified using the PDXL–2 software package (Rigaku, Tokyo, Japan) using the Powder Diffraction File database (PDF–2, International Center for Diffraction Data, 2011).

Images of nanoparticles were obtained using a transmission electron microscope with an autoemission cathode, an energy Ω filter, and a Koehler lighting system (Zeiss Libra 200FE, Oberkochen, Germany).

The size distribution by volume was studied by dynamic light scattering (DLS), the zeta potential was determined by electrophoretic light scattering (ELS) using a Zetasizer Ultra (Malvern Instruments Ltd., Malvern, United Kingdom).

**Figure 2:**
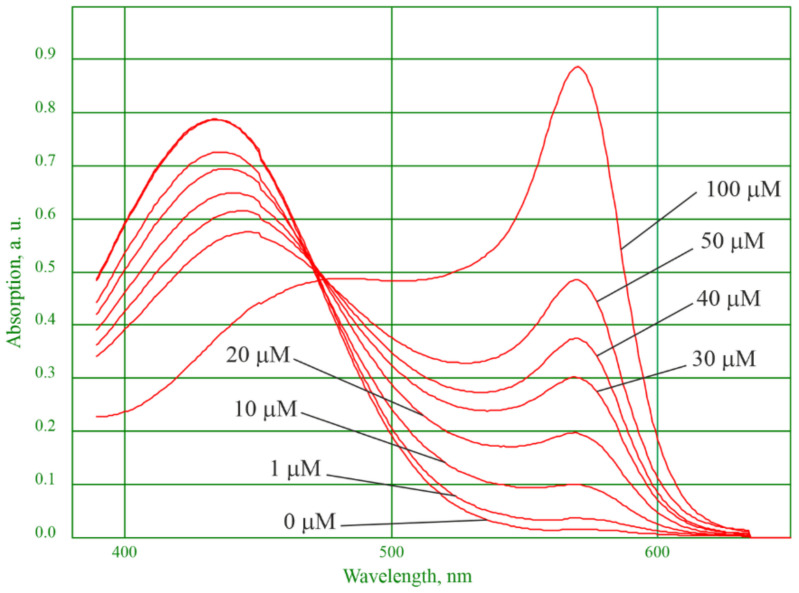
Calibration spectra with different concentrations of the gadolinium cation in the XO indicator.

Gadolinium cation content was determined by calibration. 0, 10, 100, 200, 300, 400, 500, 1000 μL of 1 mM gadolinium nitrate solution was added to a 25 mL flask and brought to 10 mL with xylenol orange (XO) aqueous solution in a concentration of 0.025 mg/mL. After 5 min, the spectra were recorded in the 350–650 nm wavelength range (Figure 2).

As it follows from Figure 2, the gadolinium cation content can be calibrated by two peaks at 433 and 570 nm.

For practical reasons, we performed a calibration built at a wavelength of 570 nm. There were two rationales for this. First, at this wavelength, the correlation coefficient is higher. Second, the investigated samples containing iron(II) and iron(III) cations showed that the peak at a wavelength of 433 nm deforms due to interaction of the XO indicator with iron cations. The peak at a wavelength of 570 nm adequately describes the content of the gadolinium cation in the presence of iron cations.

When measuring, an aliquot of 100 μL or 1 mL of the sample was taken depending on the expected gadolinium content, brought to 10 mL with XO solution containing 0.025 mg/mL, and the absorption spectrum was recorded in the wavelength range between 350 and 650 nm. The gadolinium content was determined by optical density at a wavelength of 570 nm. The result was recalculated accounting for the dilution.

The content of free gadolinium cation was determined in precipitated and ready-made mineralized samples calculated for stoichiometry of gadolinium ferrate and trigadolinium pentairon(III) oxide. For this, 50 mg of the sample was redispersed in 10 mL of distilled water, then precipitated by centrifugation at a speed of 4200 rpm for 5 min. A 1 mL aliquot was taken from the obtained supernatant and analyzed for the gadolinium cation compound.

The total gadolinium content was also determined in precipitated and freshly prepared mineralized samples calculated for the stoichiometry of gadolinium ferrate and trigadolinium pentairon(III) oxide. For that purpose, 50 mg of the sample was dissolved in 5 mL of concentrated nitric acid. The sample volume was brought to 10 mL with water, an aliquot of 100 μL was taken, which was analyzed for the gadolinium cation.

The static magnetic characteristics of GF/TPO NPs were obtained in air at a temperature of 25 °C using a Lake Shore 7410 vibration magnetometer (Lake Shore Cryotronics Inc., USA).

**Figure 3:**
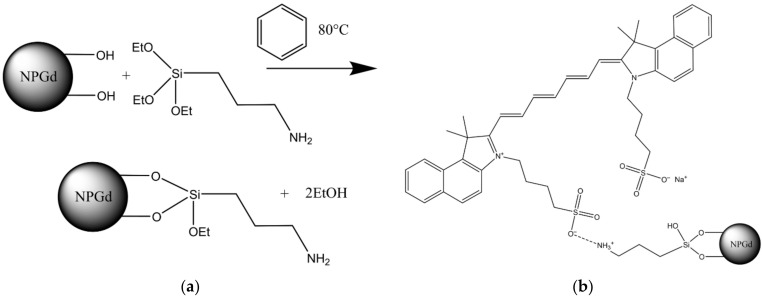
GF/TPO NP modification scheme: (**a**) Amino-spacer synthesis scheme; (**b**) coordination-ionic immobilization of ICG on an amino-spacer.

Laboratory amination of GF/TPO NP surface was carried out using previously described technique [24]. In brief, 2 g of NPGd, 23.75 mL of dry benzene and 1.25 mL of 3–aminopropyltriethoxysilane (APTES) were placed in a round-bottom 50 mL flask. The reaction mixture was heated for 2 h at 80 °C. The heating was performed with a thermostatic cell connected to a LT–105a liquid circulation thermostat (LOIP, St. Petersburg, Russia). Excess APTES was removed via repeated washing with dry chloroform using magnetic separation. The final washing was performed with ethanol. During the reaction and washing, the reaction mixture was intensively stirred using a magnetic stirrer. The resulting product was lyophilized at a temperature of −50 °C and at an absolute pressure of 3 Pa in a VaCo 2 freeze dryer (ZirBus, Germany) for 48 h. The synthesis scheme is shown in Figure 3a.

The total content of amino groups in the samples was determined by back titration. For this, 1.0 mL of 0.1 N hydrochloric acid was added to the aminated GF/TPO NPs and left for 15 min to react with amino groups, with intermittent shaking. The suspension thus treated was centrifuged for 5 min at a speed of 3000 min^−1^. The supernatant was titrated using the acid–base indicator methyl orange (0.1 N) and with alkali (NaOH). The total number of amino groups was calculated from the amount of alkali.

The number of available amino groups was determined by chemisorption of the fluorescent dye indocyanine green (ICG). ICG was chemisorbed from aqueous solution. Upon completion of the chemisorption process, the amount of adsorbed fluorophore was determined. 10 mL of 0.1 N alkali was added to the washed precipitate, and the desorption reaction was carried out for 15 min. After that, the colloid was centrifuged and the supernatant was taken by 1 mL portions. The latter was analyzed for fluorophore content on a spectrophotometer (Unico 2802s, Unico, Franksville, WI, USA) at a wavelength of 700 nm.

ICG is a cyanine dye used for fluorescent angiography in ophthalmology. In addition, it is used for determining cardiac output, hepatic function, liver and gastric blood flow. It has a peak spectral absorption at about 800 nm. This value falls within the biological tissues transparency band, which makes it very convenient to use this fluorophore in vivo.

The ICG absorption and fluorescence spectra lie in the near-infrared region. Both are highly dependent on the solvent used as well as on its concentration. ICG absorbs mainly between 600 and 900 nm, and emits fluorescence between 750 and 950 nm. The large overlap of the absorption and fluorescence spectra leads to a pronounced reabsorption of the ICG fluorescence itself. The fluorescence spectrum is very wide. Its maximum values are 810 nm in water and 830 nm in blood [25].

ICG was immobilized on the surface of GF/TPO NPs in the following way. As such, 1 mL of distilled water and 1 mL of ICG solution in a concentration of 1 mg/mL were added to 50 mg of the pre-aminated GF/TPO NPs suspension in 2 mL of water. Chemisorption was carried out on an LS–220 orbital shaker for 2 h at a 300 min^−1^ stirring speed. The reaction was carried out in 15 mL polypropylene tubes. The resulting suspension was centrifuged for 5 min at a speed of 3000 min^−1^. The centrifugate was washed five times with distilled water using centrifugation. The scheme of coordination ionic immobilization of ICG is shown in Figure 3b. In the pilot experiments, we tested the strength of ICG immobilization on aminated nanoparticles using such solvents as distilled water, 0.9% sodium chloride, and Krebs–Henseleit buffer consisting of the following components (in mmol/L): NaCl, 118.5; KCl, 4.7; NaHCO_3_, 25; KH_2_PO_4_, 1.2; MgSO_4_, 1.2; glucose, 11; and CaCl_2_, 1.5 [26]. Only trace amounts of ICG were detectable in the solvent over the period of several months. In order to provide better characterization of putative heart targeting ability of GF/TPO NPs, along with ICG we have used another fluorescent label, that is, fluorescein isothiocyanate (FITC). FITC has been covalently immobilized on the surface of GF/TPO NPs.

Colloidal stability of native and ICG-doped GF/TPO NPs in aqueous suspensions was determined by measuring time-dependent changes in light absorbance of nanoparticle suspensions at a wavelength of 300 nm over the period of 2 h.

### 2.3. In Vivo Biodistribution Assessment

The study of biodistribution of ICG-doped GF/TPO NPs was carried out on male Wistar SPF rats weighing 240–260 g. Animals were divided into two groups: the experimental group (*n* = 5) and the control group (*n* = 5) in order to account for the background radiation. Fluorescence radiation was assessed on the IVIS Lumina LT fluorescence imager (PerkinElmer, Waltham, MA, USA).

GF/TPO NPs suspension at a concentration of 2 mg/mL was injected into the tail vein in a volume of 1 mL. Then, 1 mL of saline solution was injected into the controls. After 30 min, the animals were euthanized with an overdose of isoflurane anesthesia. The control method of euthanasia was the removal of vital organs.

To assess biodistribution of fluorescently labeled GF/TPO NPs, the brain, heart, lungs, liver, kidneys and spleen were excised, washed in saline and examined on IVIS Lumina LT. Ex vivo images were fixed on the built-in filters of the device. Taking into account the properties of the fluorescent label, the following filters were chosen: 745 nm for absorption of radiation and 850 nm for emission.

Since the GF/TPO NPs have the largest size, while the spacer–fluorophore shell is very thin and chemically neutral, the research focused on the native biodistribution of the initial GF/TPO NPs. Biodistribution pattern of free and fluorophore-labeled GF/TPO NPs should coincide.

Biodistribution of GF/TPO NPs was also assessed using spectrophotometric determination of Fe^2+^/Fe^3+^ cations. Samples of the removed organs of laboratory animals were lyophilized at a temperature of −50 °C and at absolute pressure of 3 Pa using freeze-drying. Then the organs were weighed, placed in a conical flat-bottomed flask and filled with 20 mL of concentrated nitric acid and 20 mL of distilled water. The samples were boiled on a heating mantle for 60 min, which ensured their complete mineralization, after which the volume was brought to 100 mL. The resulting solution was analyzed by spectrophotometric method for the combined content of cations Fe^2+^ and Fe^3+^ in the presence of sulfosalicylic acid in the ammonia containing medium. Preliminary calibration was carried out using a weighed portion of carbonyl iron dissolved in a 1:1 mixture of hydrochloric and nitric acids. Concentration of sulfosalicylic acid aqueous was 20% by mass. The ammonia solution was topped up to achieve the 1:1 ratio. The solution prepared from the weighed portion (1000 mL) was taken with a pipette (1.0, 0.8, …, 0.2 mL aliquots) and placed into 100 mL flasks. Then the following substances were consecutively added: 10 mL of sulfosalicylic acid solution, ammonia solution until the substance turned yellow, and excess ammonia solution (total 25 mL). After 20 min the solutions were measured for optical density. Then preparation of solutions was repeated.

The analysis was performed on 0.5 mL portions of the tested solution. For each sample, the analysis was repeated three times. Then the obtained data were averaged. The resulted data were normalized to 1 g of dry bioassay.

### 2.4. The Study of Myocardial Ischemia–Reperfusion Injury Targeting with GF/TPO NPs

The study of myocardial injury visualization was performed in the in vivo model of regional myocardial ischemia–reperfusion in rat. Ischemia–reperfusion injury was induced in 15 male Wistar (SPF) rats weighing 320–370 g. The animals were kept in a barrier-type vivarium using individually ventilated cages in a 12-h light-dark cycle with a standard diet and drinking regimen provided.

All animals were randomized into three groups: control (*n* = 5), which were injected intravenously with saline solution of 1 mL (0.9% NaCl), group I (*n* = 5) that received intravenous administration of a suspension of magnetic nanoparticles of gadolinium ferrite with FITC as dye with a concentration of 2 mg/mL and a volume of 1 mL, and group II (*n* = 5) that received intravenous administration of magnetic nanoparticles of gadolinium ferrite conjugated with ICG with a volume of 1 mL and a concentration of 2 mg/mL. All substances were injected as intravenous bolus.

At the stage of induction of anesthesia, the animal was supplied with a 5% isoflurane mixture in oxygen-enriched air using a SomnoSuite anesthesia machine with a switch to a gas mixture containing 2–3% isoflurane, 35% oxygen and 63% room air during the surgical phase. After the start of mechanical lung ventilation through a tracheostomy (respiratory rate of 60 min^−1^, tidal volume of 2 mL/100 g body weight, CWE–SAR–830/AP, World Precision Instruments, Inc, Sarasota, FL, USA), catheterization (PE–50, Intramedic, Texas City, TX, USA) of the common carotid artery for monitoring mean arterial pressure (AP) and heart rate (HR). Throughout the experiment, the animals were kept on a thermostatic operating table that maintained body temperature at 37.0 ± 0.5 °C (TCAT–2LV; Physitemp Instruments Inc., Clifton, NJ, USA). For intravenous administration of solutions, the right femoral vein was catheterized. Throughout the entire stage of modeling myocardial ischemia–reperfusion, an electrocardiogram (ECG) was monitored in the second standard lead.

After 30 min of the stabilization period, left-sided thoracotomy was performed in the fourth left intercostal space. A thin polypropylene ligature (6–0) with an atraumatic needle was placed under the left coronary artery (LCA). The ends of the ligature were passed through a 7–8 cm section of a polyethylene tube (PE–90, Intramedic, Texas City, TX, USA), an occluder. Myocardial ischemia was initiated 30 min after the end of surgical procedures and stabilization of hemodynamic parameters by moving the occluder downward and applying a clamp to the occluder. After 30 min of myocardial ischemia, the clamp was removed to restore blood flow for 120 min.

**Figure 4:**
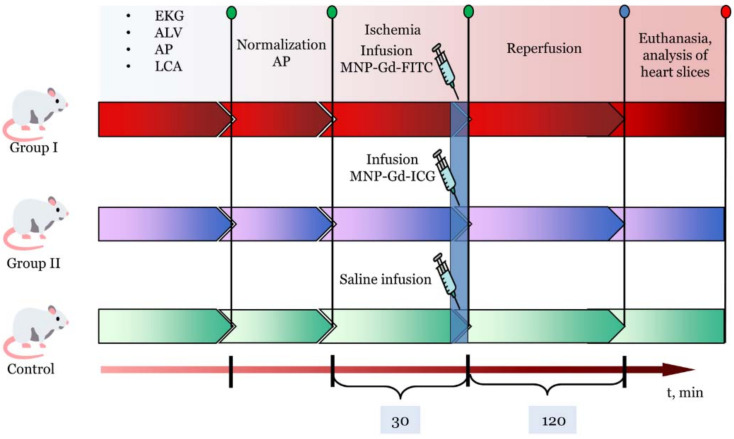
Scheme of an acute experiment on three different groups.

Registration of hemodynamic parameters was carried out immediately before 30 min of occlusion, 15 min of ischemia, 40, 90 and 120 min of reperfusion. Exclusion criteria: AP < 50 mm Hg and/or heart rate < 300 at any time during the experiment. At the end of the experiment, the animals were euthanized with subsequent determination of the size of the infarction. The study design is shown in Figure 4.

The fluorescence imaging of the sections was carried out on an IVIS Lumina III fluorescence imager (Perkin Elmer, Waltham, MA, USA) and the operating modes which were selected taking into account the dyes used in the experiment, for example; built-in filters 465–GFP were selected for the FITC dye, and 745–ICG for the ICG. The intensity of fluorescent radiation of heart slices was quantified using the built-in Living Image 4.5.5 software (PerkinElmer Inc., Waltham, MA, USA).

## 3. Results and Discussion

### 3.1. Physicochemical Properties

The study resulted in a new GF/TPO NPs synthesis method based on the hydroxides of gadolinium(III), iron(II), iron(III) co-precipitation and mineralization under hydrogen atmosphere at a temperature of 500 °C.

The specific surface area determined by the BET method by low temperature nitrogen adsorption for gadolinium ferrate was 42 m^2^/g, for trigadolinium pentairon(III) oxide it was 66 m^2^/g.

**Figure 5:**
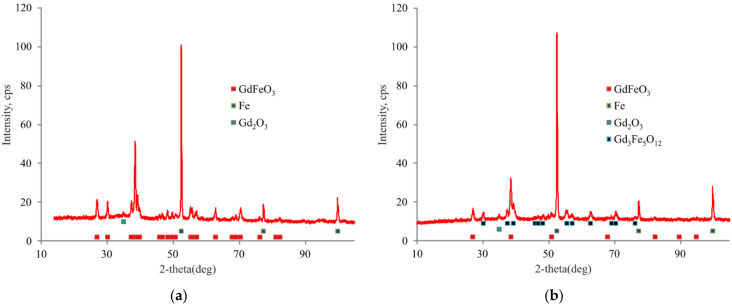
X-ray diffraction pattern of (**a**) an instance calculated for gadolinium ferrate and (**b**) trigadolinium pentairon(III) oxide.

A sample calculated for gadolinium ferrate contains GdFeO_3_ admixed with iron and gadolinium oxide (Figure 5a). The sample calculated for trigadolinium pentairon(III) oxide contains Gd_3_Fe_5_O_12_ admixed with gadolinium ferrate and gadolinium oxide (Figure 5b).

**Figure 6:**
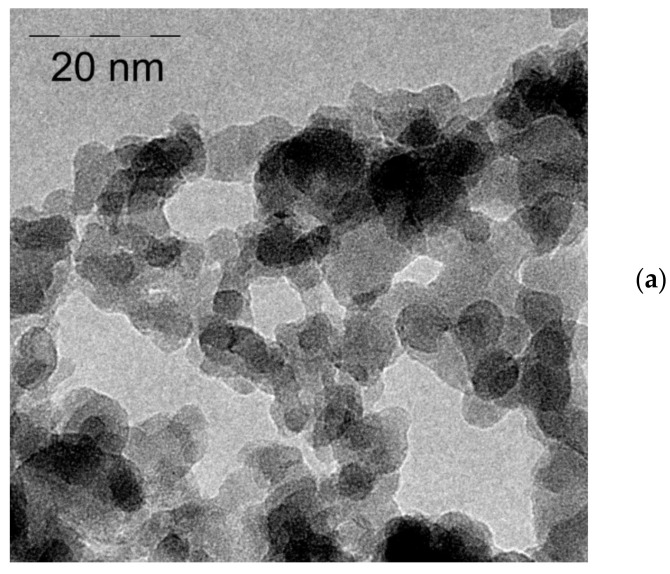

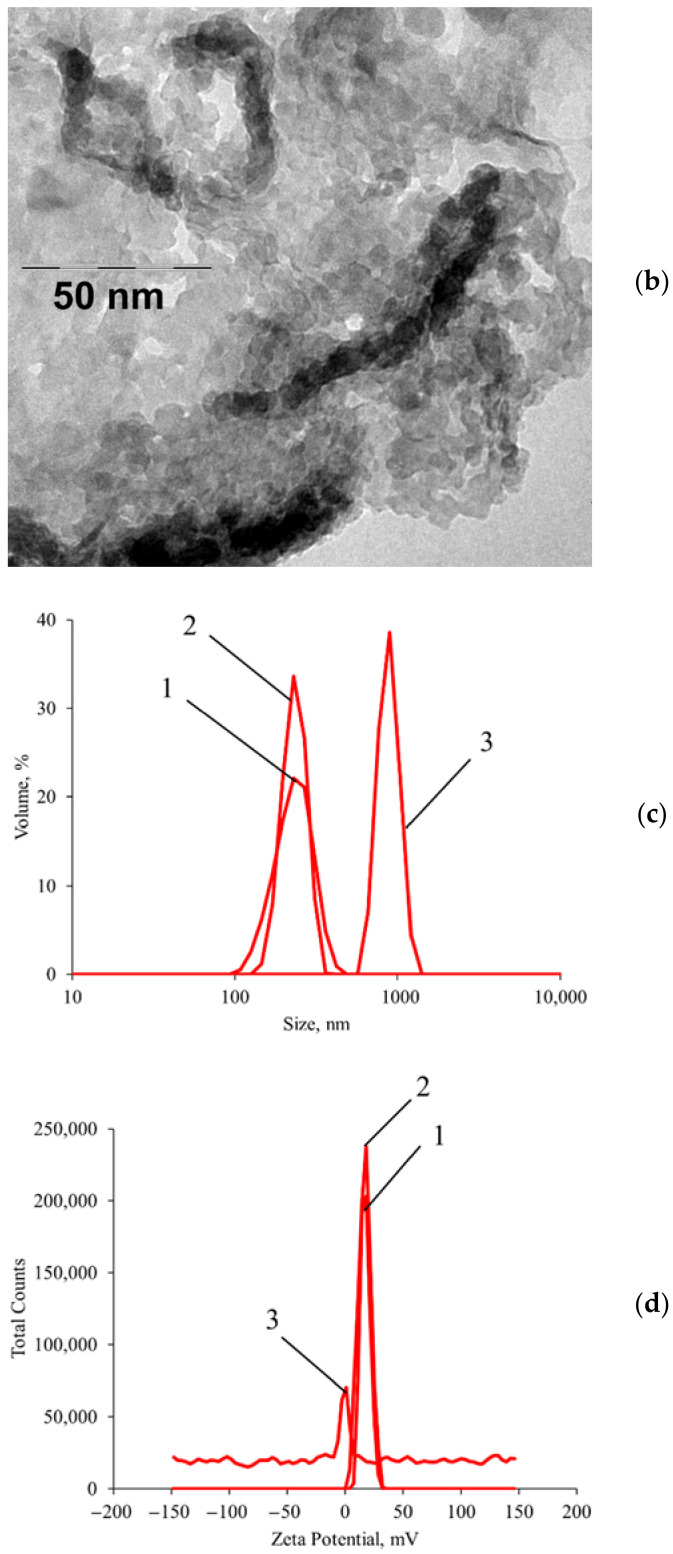
Physical properties of nanomaterials: (**a**) TEM photograph of native trigadolinium pentairon(III) oxide; (**b**) TEM photograph of trigadolinium pentairon(III) oxide with immobilization of ICG on an amino-spacer; (**c**) Size distribution; (**d**) Zeta potential; (1) Gd_3_Fe_5_O_12_; (2) Gd_3_Fe_5_O_12_–NH_2_; (3) Gd_3_Fe_5_O_12_–NH_2_–ICG.

Figure 6a shows a TEM photograph of unmodified trigadolinium pentairon(III) oxide with the average size of 10 nm. Figure 6b shows TEM image of GF/TPO NPs at higher concentration with immobilization of ICG on an amino-spacer. The average hydrodynamic diameter of Gd_3_Fe_5_O_12_, determined by the DLS method, was approximately 230 nm (Figure 6c). The aminated sample has a similar value. This fact indicates that, in an aqueous solution, the initial crystallites, 10 nm in size, form associates, and treatment with an aminating agent gives a very thin shell, which practically does not affect their size. ICG immobilization increases the size of NPs to 800 nm. This, apparently, is due to the change in the zeta potential of nanomaterials from 20 mV (Figure 6d) to almost zero. In addition, formation of bonds between sulfonate moieties of ICG molecules immobilized on neighboring nanoparticles could partly account for the enhanced nanoparticle aggregation. In this study, we have not used protective capping of sulfonate sites in order to prevent bridging.

As shown in Table 2, the gadolinium content in non-mineralized and mineralized samples is close to the theoretically calculated value. The content of free gadolinium is at the method sensitivity level. In other words, there is practically no free gadolinium in the samples, which allows for the theoretical possibility of using the obtained nanoparticles in vivo.

**Figure 7:**
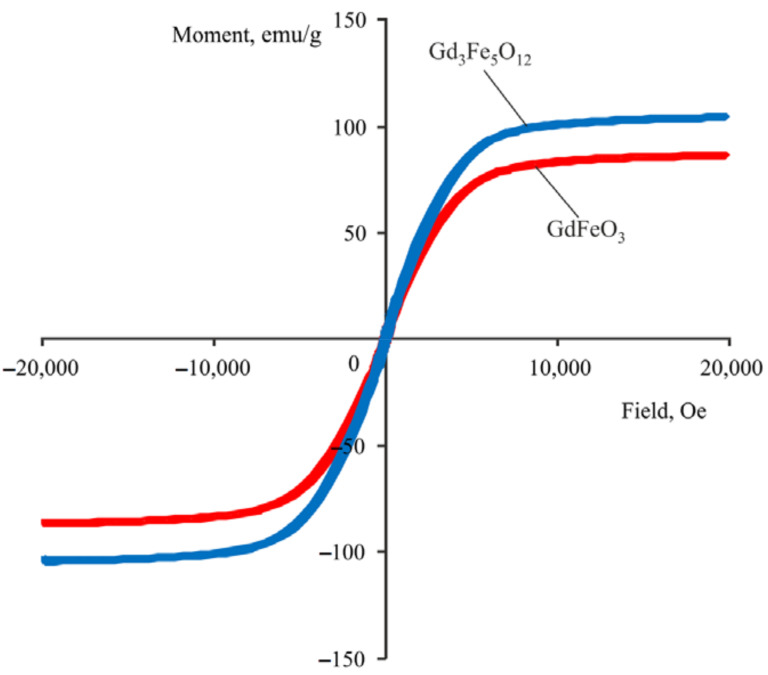
Magnetization reversal curves of GF/TPO NP samples.

Magnetization reversal curves for gadolinium ferrate and trigadolinium pentairon(III) oxide are shown in Figure 7. Magnetic characteristics of the samples (Table 3), obtained from the magnetization reversal curves, indicated that the gadolinium ferrate sample has the saturation magnetization equal to 86 emu/g, while trigadolinium pentairon(III) oxide is characterized by a larger value equal to 105 emu/g. This result exceeds the value of the saturation magnetization of a single crystal magnetite, which is equal to 94 emu/g. The coercive force of the samples appeared to be equal to 100 and 30 Oe, respectively. Since the best magnetic characteristics were obtained for trigadolinium pentairon(III) oxide, further experiments were carried out with it.

In the aminated samples, the total amount of amino groups was 0.84 mmol/g, while the amount of available amino groups was 0.0042 mmol/g.

The ICG was immobilized onto aminosilane spacer in an ion-coordination manner. The studies have shown that the sulfo group included in ICG forms a rather strong ionic bond with the amino group, and does not hydrolyze. The amount of fluorophore immobilized in this way was 3.72 mg per gram of GF/TPO NPs.

**Figure 8:**
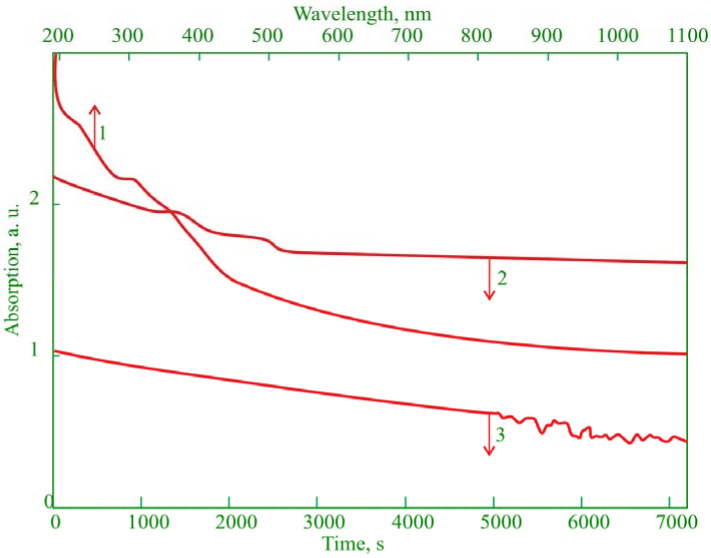
Colloidal stability of GF/TPO NP samples. Time-dependent changes in light absorbance of nanoparticle suspensions at a wavelength of 300 nm over the period of 2 h were determined. (1) absorption spectrum of the native GF/TPO NPs; (2) time-dependent changes in the absorbance of native GF/TPO NP suspension at λ = 300 nm over 2 h; (3) time-dependent changes in the absorbance of ICG-modified GF/TPO NP suspension at λ = 300 nm over 2 h.

The data on colloidal stability of both native and ICG-modified GF/TPO NPs are presented in Figure 8. The suspension of native GF/TPO NPs demonstrated reduction in absorbance during first 40 min followed by its stabilization, providing evidence for sedimentation of larger particles over the period of 40 min. The remaining small particles were stable. The suspension of GF/TPO NPs with immobilized ICG was characterized by gradual decrease in absorbance for 2 h. After approximately 80 min, some fluctuations were observed, possibly indicating the formation of larger particles.

### 3.2. Biodistribution

**Figure 9:**
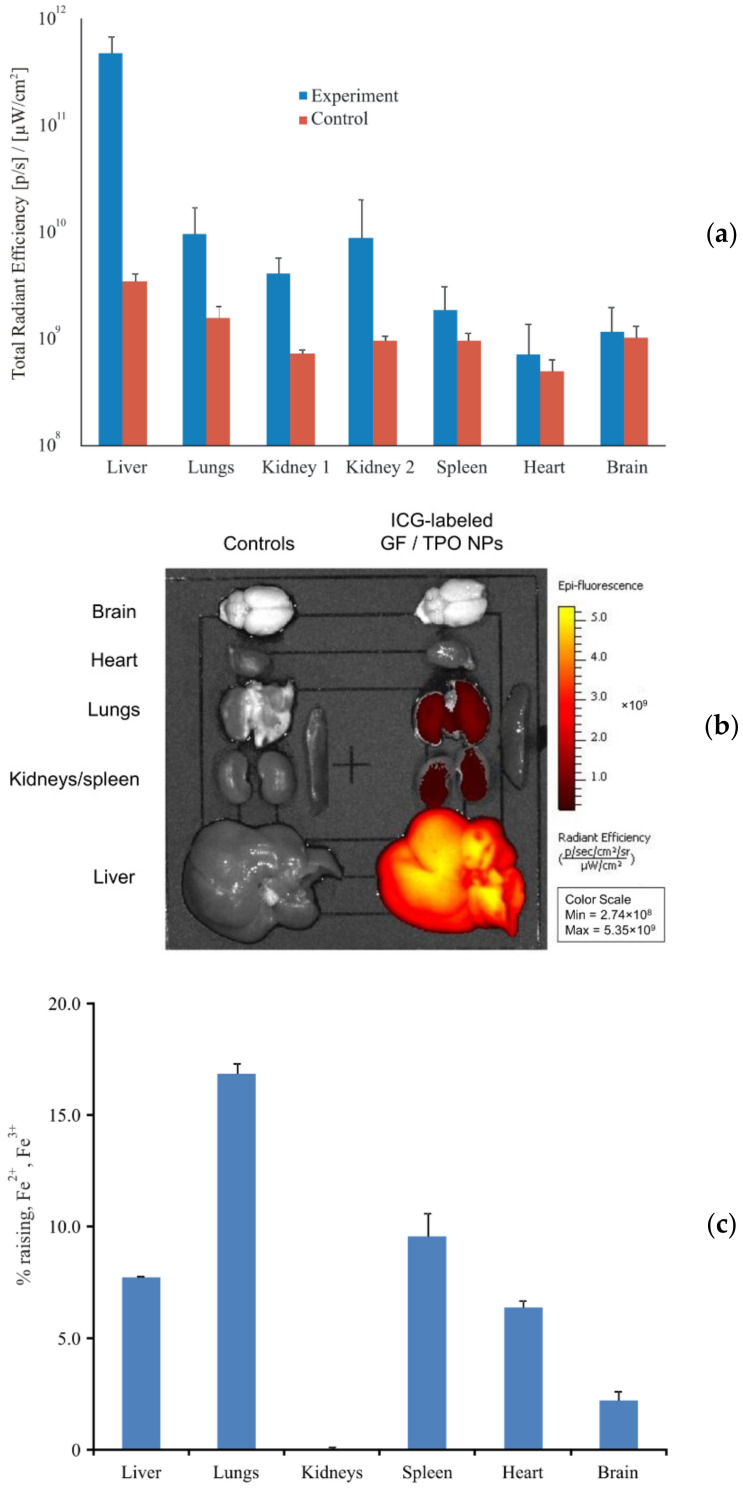
The results of in vivo biodistribution studies. ICG-labeled GF/TPO NPs in suspension at a concentration of 2 mg/mL were injected into the tail vein of rats in a volume of 1 mL (*n* = 5). 1 mL of saline solution was injected into the controls (*n* = 5). After 30 min, the animals were euthanized followed by harvesting of main organs. The extent of nanoparticle accumulation in different organs was evaluated by either fluorescent imaging or quantification of tissue iron content: (**a**) Biodistribution of GF/TPO NPs with ICG; (**b**) Representative images of organs harvested from control animals and animals injected with ICG-labeled GF/TPO NPs. On the left row, the organs harvested from controls are shown, while on the right row the organs obtained from experimental group are demonstrated; (**c**) An increase in the quantity of iron cations in organs in relation to the control.

The study of native biodistribution performed using a fluorescent label showed a predominant accumulation of GF/TPO NPs in three organs: liver, lungs, and kidneys (Figure 9a). Accumulation in the spleen, heart and brain as compared with the control value was minimal. Accumulation in the liver and kidneys indicates a drug metabolism pathway. Accumulation in lungs is apparently associated with the presence of a large number of small vessels, in which GF/TPO NPs are retained.

The study of native biodistribution of iron content in organs showed slightly different results (Figure 9b). As in the case of fluorescence biodistribution studies, large amounts of GF/TPO NPs were retained in the lungs, while GF/TPO NPs were also found in the liver. A significantly smaller amount of GF/TPO NPs is due to the fact that the mass of iron is brought to the mass of the organ, and the liver is a rather massive organ. An interesting fact is that in the kidneys no excess of the iron level over the endogenous level was ever found. An increase in iron content was also found in the spleen, heart, and brain as compared to the control value.

### 3.3. Myocardial Ischemia–Reperfusion Injury Targeting with GF/TPO NPs

**Figure 10:**
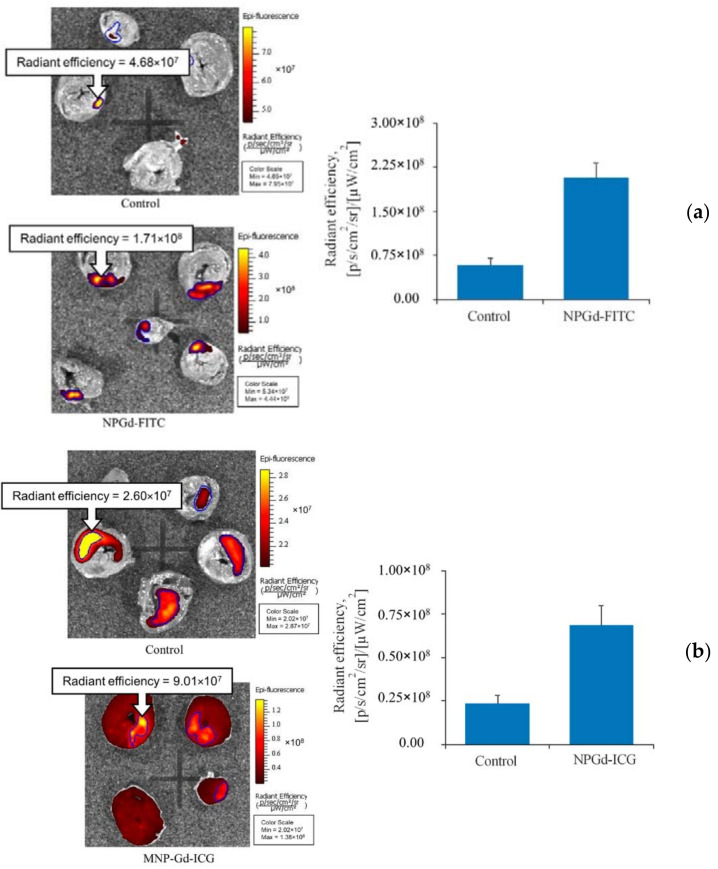
Heart targeting by GF/TPO NPs in the setting of ischemia–reperfusion injury. The animals were randomized into three groups: (1) controls (*n* = 5) received i.v. infusion of saline, (2) NPGd–FITC (*n* = 5) were injected with FITC-labeled GF/TPO NPs at a dose of 2 mg/mL; (3) NPGd–ICG (*n* = 5) were injected with ICG-labeled GF/TPO NPs at the same dose. All injections were performed just after 30-min myocardial ischemia. After 120 min of reperfusion, the animals were euthanized, the hearts were excised and sliced into 4 or 5 transverse slices. Comparison of the sample fluorescence intensity in the area of ischemia–reperfusion injury is shown, as well as the representative images of heart slices: (**a**) NPGd–FITC; (**b**) NPGd–ICG.

To explore the potential targeting ability of GF/TPO NPs toward myocardial ischemia–reperfusion injury, we studied the accumulation of GF/TPO NPs labeled with FITC and ICG in the area of infarction versus normal myocardium [27]. Two fluorophores operating in different wavelength ranges provide a more accurate imaging picture. The scheme of fluorophore immobilization and the properties of nanoparticles with immobilized FITC are similar to those with ICG. The intensity of fluorescence in the area of ischemia–reperfusion injury versus normal myocardium after injection of FITC- and ICG-labeled magnetic nanoparticles of gadolinium ferrite is shown in Figure 10a.

In this study, there is a staining of the ICG infarction zone, the fluorescence of which significantly exceeds the fluorescence of the zones in the control group. This suggests that the synthesized multilayer nanoparticles can be used in animal studies to delineate the area of myocardial infarction and to determine its size.

For nanoparticles with ICG (NPGd–ICG), the distribution has a different form (Figure 10b). Staining occurred not only in the area of damage, but throughout the entire area of the sections, possibly due to breaks of bonds in the nanoparticles, after which the ICG circulated throughout the blood system. Apparently, this is due to various methods of immobilization of fluorophores on the spacer, that is, coordination ionic immobilization for ICG and covalent immobilization for FITC. As expected, the covalent immobilization method binds the fluorophore more tightly, preventing it from being cleaved in the circulation.

## 4. Conclusions

The study describes a new technique for the synthesis of gadolinium ferrate and trigadolinium pentairon(III) oxide nanoparticles, including precipitation of a mixture of iron(II), iron(III) and gadolinium(III) hydroxides under the action of ammonium hydroxide, followed by lyophilization and mineralization in a hydrogen atmosphere. As a result, magnetic nanoparticles with a developed surface were obtained, allowing immobilization of fluorophores. The trigadolinium pentairon(III) oxide nanoparticles showed high magnetization in combination with a low coercive strength, which makes it possible to use them for magnetically controlled targeted drug delivery. The immobilization of the fluorescent dye indocyanine green on the surface of magnetic particles modified with amino groups was carried out, which made it possible to study the biodistribution of nanoparticles in rats. Investigation of biodistribution using a fluorescent label reliably demonstrated that the nanoparticles predominantly accumulate in the liver, lungs and kidneys. The obtained nanomaterials are targeted toward the area of myocardial infarction and thus can be used for theranostic applications.

## Figures and Tables

**Table 1:** Proportion of reagents in the reaction mixture.

Reagent	Gadolinium Ferrate	Trigadolinium Pentairon(III) Oxide
Distilled water, mL	100.00	100.00
Gadolinium nitrate(III), g	1.21	0.72
Iron sulfate(III), g	2.26	2.26
Iron sulfate(II), g	2.00	2.00
Ammonium citrate, g	0.25	0.25

**Table 2:** Quantity of total and free gadolinium in samples.

Sample Type	Quantity Gd^3+^, % Mass	
	Non-Mineralized	Mineralized
Calculated for gadolinium ferrate, redispersed (free gadolinium)	0.103	0.341
Calculated for trigadolinium pentairon(III) oxide, redispersed (free gadolinium)	0.350	0.291
Calculated for gadolinium ferrate, dissolved in HNO_3_ (total gadolinium)	28.877	30.537
Calculated for trigadolinium pentairon(III) oxide, dissolved in HNO_3_ (total gadolinium)	29.847	30.352

**Table 3:** Magnetic characteristics of nanoparticles.

Sample Type	Saturation Magnetization, emu/g	Coercive Force, Oe
Gadolinium ferrate	86	100
Trigadolinium pentairon(III) oxide	105	30

## Data Availability

Not applicable.
